# Give the community the tools and they will help finish the job: key population‐led health services for ending AIDS in Thailand

**DOI:** 10.1002/jia2.25535

**Published:** 2020-06-09

**Authors:** Ravipa Vannakit, Surang Janyam, Danai Linjongrat, Pongthorn Chanlearn, Satayu Sittikarn, Supabhorn Pengnonyang, Rena Janamnuaysook, Krittaporn Termvanich, Reshmie Ramautarsing, Nittaya Phanuphak, Praphan Phanuphak

**Affiliations:** ^1^ Independent Researcher Bangkok Thailand; ^2^ Service Worker In Group Foundation (SWING) Bangkok Thailand; ^3^ Rainbow Sky Association of Thailand (RSAT) Bangkok Thailand; ^4^ Mplus Foundation Chiang Mai Thailand; ^5^ CAREMAT Chiang Mai Thailand; ^6^ Thai Red Cross AIDS Research Centre Bangkok Thailand

**Keywords:** community, differentiated care, health systems, HIV care continuum, key and vulnerable populations, task shifting

Time is running out for countries to end AIDS by 2030. Success will require putting “fast‐track” solutions in the hands of those who can make the greatest impact – the community. Progress in engaging communities in the planning and delivery of health services, which the WHO recommended as a task‐shifting strategy over a decade ago [1], has been painfully slow. Task shifting HIV service delivery to the affected community will broaden options for service delivery and extend the reach of services among those in need [2].

The *Key Population‐led Health Services (KPLHS)* model was established in Thailand in 2015 to demonstrate how task shifting can be realized through delivering HIV and health services that would normally be delivered by medical professionals in health facilities, by lay providers who are members of the key population communities. In the context of the Thai HIV epidemic, the affected communities or key populations (KP) comprise men who have sex with men (MSM), transgender women (TGW), sex workers (SW) and people who inject drugs (PWID) who contributed to two‐thirds of new HIV cases during 2015 to 2019 [3].

The KPLHS approach was proposed by grass root MSM, TGW and SW communities. It is a model that has demonstrated feasibility, acceptability and affordability of KP‐led service delivery. This optimizes KP contextual knowledge and connections to help navigate hardest‐to‐reach and at‐risk individuals to where essential health and HIV services can be obtained. These are designed and co‐delivered by the KP community, in close collaboration with the public health sector, to ensure services are free from disrespectful care, verbal and physical abuse, and outright denial of care due to stigma and discrimination which often characterise conventional health care settings [4].

The design of the service package is *needs‐based, demand‐driven, and client‐centred*. For example, a service package designed for TGW integrates gender affirming care with sexual health service to address common health concerns prioritized by TGW [5], while for SW, legal assistance and out‐of‐school education are co‐located in sexual health clinics to provide both social and clinical services highly needed among this community. KPLHS follows three principles: (i) KP‐friendliness: that is, non‐stigmatizing and confidential; (ii) accessibility: that is, flexible service hours, low or no cost, and geographically close to KP’s workplaces and gathering venues; and (iii) quality: that is, adhering to national regulations and standards for health service delivery.

KPLHS supports Thailand’s National AIDS Strategies to enhance uptake of HIV services along the Reach‐Recruit‐Test‐Treat‐Retain cascade. Comprehensive KPLHS services are set out in Figure 1. All services are delivered in community settings by trained KPLHS lay providers, tailored to the needs of each KP community and linked closely with the public health sector.

**Figure 1:**
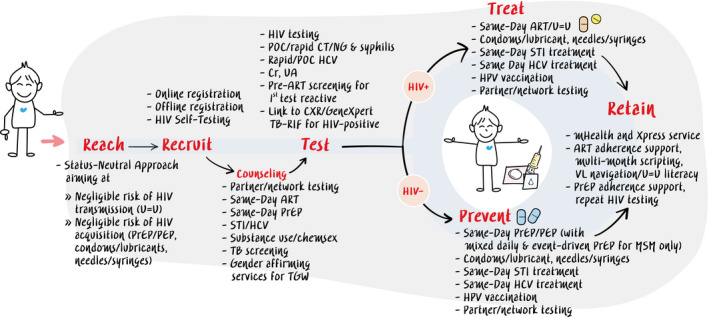
Key population‐led health services flow along the Reach‐Recruit‐Test‐Treat‐Prevent‐Retain cascade. ART, antiretroviral therapy; Cr, creatinine; CT, *Chlamydia trachomatis*; CXR, chest x‐ray; HCV, hepatitis C virus; HPV, human papillomavirus; mHealth, mobile health; MSM, men who have sex with men; NG, *Neisseria gonorrhoea*; PEP, post‐exposure prophylaxis; POC, point‐of‐care; PrEP, pre‐exposure prophylaxis; STI, sexually transmitted infection; TB, tuberculosis; TGW, transgender women; U=U, undetectable equals untransmittable; UA, urine analysis; VL, viral load; Xpress, express.

KPLHS lay providers are equipped through systematic training, mentoring, coaching and certification to provide comprehensive HIV and sexually transmitted infection (STI) prevention and treatment services [6], including point‐of‐care HIV/STI testing, pre‐ and post‐exposure prophylaxis (PrEP/PEP), treatment service linkages, and case management support. Services are provided in an express fashion aiming at service completion within the same day to minimize leakage in the HIV/STI service cascade. The public health sector supports quality assurance, accreditation, linkages to treatment and harmonization of data monitoring and reporting systems.

KPLHS takes advantage of the widespread use of mobile phones and social media platforms to enhance HIV service uptake and retention. KP communities have developed online tools to map their networks to differentiate outreach activities based on case finding results and to link those who are reached online to offline services through online booking. Assistance for HIV self‐testing in community settings and online supervision are provided.

Involving KP communities in HIV service provision is efficient for preventing HIV infection, loss to follow up and earlier treatment initiation. Data for 2018 show that KPLHS has enabled early diagnosis with a median CD4 count at diagnosis of 388 cells/mm^3^ [7], compared to 192 cells/mm^3^ in public health facilities [8]. It has improved treatment outcomes. 84.3% (730/866) of newly diagnosed HIV‐positive clients in KPLHS sites were successfully linked to antiretroviral therapy initiation and 95.6% (537/562) tested for viral load had viral load suppression [7]. It has facilitated the uptake of PrEP among KP. 36% of 7670 HIV‐negative clients at risk for HIV infection who were offered PrEP, accepted it [9]. These metrics have been instrumental in gaining the acceptance of the Thai HIV policy community and medical professionals.

Evidence‐based advocacy, publication in peer‐reviewed journals and concerted policy dialogue, involving academics and KP community leaders led to the government removing regulatory barriers for lay provider testing and increasing domestic financing through social contracting mechanisms [10]. Attitudes of medical health professionals towards lay providers have changed to become more accepting and supportive.

Sustaining this model involves institutionalizing: (i) technical capacity of KP service providers; (ii) a quality assurance system; and (iii) KPLHS inclusion in the overall universal health care system and budgets. It is critical to invest more in professionalizing KPLHS providers to enhance their technical skills and reputation which are essential to enable government funding of KPLHS.

Further work is needed in Thailand to enable scaling up of KPLHS to end AIDS in the shortest possible timeframe. Competing public health priorities, including emerging infectious diseases will inevitably divert resources from HIV/AIDS and put additional pressure on the functioning of health systems. At this juncture, it is necessary to emphasize the significant contribution that community can make in health system strengthening overall.

The KPLHS model can be adapted to different priority populations, public health priorities and country contexts, particularly where social stigma and discrimination associated with health issues undermine access to health care settings. The model is currently being adapted for the PWID community in Thailand but it is at an early stage of implementation. It has a focus on integrated HIV/hepatitis C testing and treatment. Adaptation to other country contexts will depend on building the credibility, capacity and commitment of the KP community to take on this approach and for governments to follow the science to implement task shifting at scale.

## COMPETING INTERESTS

All authors declare no competing interests.

## AUTHORS’ CONTRIBUTIONS

NP and RV developed the conceptualization and design of the Viewpoint. RV was the principal author of the first draft. PP and NP provided critical guidance, input and subsequent revisions. KT developed the figure illustrating KPLHS service flow. SJ, DL, PC, SS, SP, RR and RJ provided inputs and essential references. All authors have reviewed and approved the final article.
